# Recent advances and current challenges in suture and sutureless scleral fixation techniques for intraocular lens: a comprehensive review

**DOI:** 10.1186/s40662-024-00414-0

**Published:** 2024-12-30

**Authors:** Han Sun, Caixia Wang, Hong Wu

**Affiliations:** Department of Ophthalmology, The Second Hospital of Jilin University, Changchun, China

**Keywords:** Aphakia, Intraocular lens implantation, Intrascleral fixation, Modified Yamane technique, Sutureless fixation, Transscleral suture fixation

## Abstract

Over the past two decades, both suture and sutureless techniques for scleral fixation of intraocular lenses have seen significant advancement, driven by improvements in methodologies and instrumentation. Despite numerous reports demonstrating the effectiveness, safety, and superiority of these techniques, each approach carries with it its own drawbacks, including an elevated risk of certain postoperative complications. This article delves into various surgical techniques for scleral fixation of posterior chamber intraocular lenses, discussing their procedural nuances, benefits, drawbacks, postoperative complications, and outcomes. Furthermore, a comparative analysis between suture and sutureless fixation methods is presented, elucidating their respective limitations and associated factors. It is hoped that this comprehensive review will offer clinicians guidance on how to individualize procedural selection and mitigate surgical risks, and thus achieve optimal visual outcomes. This review will also endeavor to provide guidance for future advancements in intraocular lens fixation techniques.

## Background

The primary indications for intraocular lens (IOL) fixation include aphakia following lens extraction, trauma-induced aphakia, dislocation or subluxation of the lens (whether congenital, secondary, or traumatic), displacement or subluxation of a previously implanted IOL, and the necessity for IOL replacement due to complications. When IOL fixation is required, several techniques are available. If sufficient capsular support remains, options include implanting a posterior chamber IOL (PCIOL) within the capsular bag or positioning a PCIOL in the ciliary sulcus, anterior to the anterior capsule. In the absence of the capsular bag but with the presence of the iris, alternatives such as an iris-claw IOL (IC-IOL) and suturing a PCIOL to the iris are viable. When both the iris and capsular bag are absent, the options extend to suturing a PCIOL to the sclera or utilizing a sutureless intrascleral fixation technique.

The optimal position for IOL implantation is in the capsular bag [1, 2]. Nonetheless, defects such as an incomplete capsular bag or inadequate residual capsular support may result in IOL implantation failure, in the wake of trauma, surgery, or other factors [2].

IC-IOL is designed to secure the lens by embedding iris tissue into grooves at the haptic ends, allowing for placement in either the anterior or posterior chamber [3]. The most commonly used lens in this category is the Artisan iris-claw IOL, which is particularly advantageous for patients lacking capsular support [4]. However, it is not recommended for patients with pathological mydriasis, extensive iris defects, ischemic vitreoretinal conditions, or uveitis [3, 5]. Potential complications include corneal endothelial cell loss, iris depigmentation, pupillary block, and fundus pathologies [6–8]. Additionally, studies have suggested that positioning these IOLs in the anterior chamber may elevate the risk of corneal endothelial loss [9]. For eyes implanted with IC-IOL, pupil shape and reactivity to light or pharmacological agents may be affected [10]. Bellucci et al. demonstrated that IC-IOL can restrict pupillary dilation when stimulated pharmacologically although this effect is less pronounced under natural light conditions [10]. Additionally, the IC-IOL requires a larger incision, which can result in increased postoperative astigmatism and a higher risk of wound leakage during the early postoperative period [11, 12].

Transscleral or intrascleral fixation of the PCIOL is often preferred as the primary treatment for aphakic eyes without adequate capsular support [13]. This preference is due to the IOL's proximity to the natural lens position, which avoids potential complications associated with anterior chamber IOL implantation, such as damage to the anterior chamber angle, corneal endothelium, and iris [14, 15].

The fixation of PCIOL has mainly gone through three stages: traditional transscleral PCIOL suture fixation, knotless PCIOL fixation represented by zigzag-shaped suture (Z-suture), and sutureless intrascleral IOL fixation [16–18]. Furthermore, suture fixation techniques can be divided into scleral flap and scleral flapless suture fixation [19].

The primary characteristic of the suture transscleral PCIOL fixation technique is the use of a suture knot, which, in turn, can cause complications such as suture breakage and knot exposure [19]. Several studies have reported that the rate of suture erosion or exposure in transscleral suture fixation may vary from 14.7% to 17.9% after 1 year post-surgery, with this incidence rising to as much as 73% after 2 years post-surgery [16, 20, 21]. It has been demonstrated that although the suture knot is well-buried initially in patients undergoing transscleral fixation, the knot becomes exposed as the scleral flap undergoes atrophy over time [22]. IOL dislocation due to suture breakage following transscleral fixation is a significant concern for this procedure in addition to suture knot exposure. Previous studies have indicated that there is an occurrence of IOL dislocation among patients with lens subluxation ranging from 4 to 10 years post-surgery due to suture breakage, with a range of 17% to 28% [23, 24]. These complications pose significant obstacles to the widespread adoption of this surgery. In addition, the type of sutures used in transscleral fixation, and its long-term stability are also controversial. Consequently, a novel technique has emerged for treating aphakic eyes lacking capsular support: sutureless intrascleral fixation of PCIOL [25].

The techniques of sutureless intrascleral fixation of PCIOL encompass various approaches, including preparing scleral tunnels with needle or vitreous retinal knife [26], flanged intrascleral IOL fixation with Yamane’s double-needle technique [27], transconjunctival intrascleral fixation [28, 29], and self-sealing sclera incision fixation [30, 31]. Additionally, techniques for preparing scleral flaps, such as fibrin glue-assisted intrascleral sutureless IOL fixation, are also employed [32]. Based on current reports, sutureless intrascleral PCIOL fixation presents as an effective, secure, and practical surgical method with satisfactory short-term results following postoperative observation [33]. Nonetheless, the number of cases reported in the literature is limited and the follow-up periods are short. Therefore, an extended follow-up of a larger number of cases is imperative to ascertain its long-term efficacy, safety, and stability [19].

Given the invaluable significance of scleral IOL fixation for patients with complicated lens dislocation and absent capsular support, this article provides a comprehensive review of its development and evolution. The primary goal in scleral fixation is to achieve optimal stabilization of the lens haptic and to maintain this stabilization over the long term. This review analyzes postoperative complications and their potential causes and aims to furnish ophthalmologists with a reference for selecting surgical options. By detailing the procedural nuances, benefits, and drawbacks of various methods, it offers a thorough comparison of traditional and modern approaches. The review highlights how the significant advancements in recent techniques not only provide practical advantages but also effectively address the challenges associated with earlier methods, thereby making scleral fixation more widely adopted and successful in clinical practice.

## Suture scleral fixation of IOL

### Suture scleral fixation with scleral flaps

Transscleral suture fixation is a frequently employed technique for scleral fixation which generally necessitates a substantial opening in the conjunctival tissue and the formation of a scleral flap to conceal the suture knots [19, 34]. Scleral flap suture fixation IOL can be classified into two-point and four-point fixation methods based on the number of fixation points [19].

The original technique for scleral-fixated IOL employs a two-point fixation at two symmetrical positions on the sclera [35, 36]. Two-point fixation of IOL results in postoperative instability because the principle behind it involves connecting two points to create a line [37–39]. Consequently, the incidence of IOL dislocation has been documented to be 9% in patients who have undergone scleral-fixated IOL combined with pars plana vitrectomy, IOL tilt 11%, and pupillary capture 23% [40]. These findings highlight the limitations and potential risks of two-point fixation.

The problem appears to be resolved by four-point fixation [41]. Four-point scleral suture fixation helps prevent IOL tilt and postoperative pupillary capture as well as reduce the risk of complications such as cystoid macular edema (CME) and pigmentary glaucoma [42–46]. However, this technique typically necessitates a more extensive conjunctival peel and scleral flap to cover the two external suture knots due to the four points on the sclera, resulting in excessive surgical trauma [41]. Therefore, surgeons must carefully evaluate patient characteristics and potential complications when selecting this method.

### Suture flapless scleral fixation

Traditional transscleral suture fixation involves burying the suture knots using scleral flaps, but it can give rise to complications such as exposed suture knots and endophthalmitis [22]. The technique of conjunctival incision and creating scleral flaps to cover external suture knots, however, is traumatic [34]. In cases of previous surgery or trauma, scarring of the conjunctiva and sclera may make surgery more difficult, rendering this technique impractical for cases with excessive conjunctival scarring [41].

Especially, the maintenance of the conjunctiva integrity is crucial for patients who require glaucoma surgery. Secondary glaucoma is a frequent complication for patients with spherophakia [47]. Studies have indicated that 7.7% of eyes necessitate glaucoma surgery after lensectomy due to ineffective control of intraocular pressure (IOP) [47].

Consequently, suture flapless PCIOL fixation techniques have been developed, effectively avoiding complications associated with suture knots and providing a less invasive alternative.

#### Hoffman pocket and modified technique

In response to the above challenges, Hoffman et al. proposed a method of creating scleral pockets without conjunctival dissection [48]. To create the pocket, two clear corneal incisions were made at the corneal limbus, followed by a paracentesis anterior to each incision which entered the anterior chamber [48]. This approach offers several advantages, including the preservation of conjunctival integrity, elimination of the need for scleral flap fabrication and suturing, prevention of suture knot abrasion and exposure, and reduction of the operative astigmatic effect [1, 49]. In a retrospective study conducted by Yeung et al., CME occurred in 8% of the eyes, while IOL dislocation occurred in 3% of the eyes during a 2-month follow-up period [50]. Notably, there were no reported cases of suture exposure or endophthalmitis, highlighting the effectiveness and safety of this innovative approach [50].

However, the majority of studies on the Hoffman pocket technique have been restricted to single-piece polymethylmethacrylate (PMMA) lenses or IOLs with closed-loops, and there have been few reports of its use with three-piece IOLs. Because of this, some surgeons have modified the technique and applied it to the scleral fixation of foldable three-piece IOLs [49]. The modified technique combines the advantages of scleral pocket technology with the use of a double-armed 9–0 polypropylene suture, which further reduces the possibility of suture breakage and has better long-term IOL stability [49]. This technique not only broadens the range of IOL options available for suture fixation, but also delivers a comparatively steady, secure, and uncomplicated approach for intrascleral fixation [49].

**Fig. 1:**
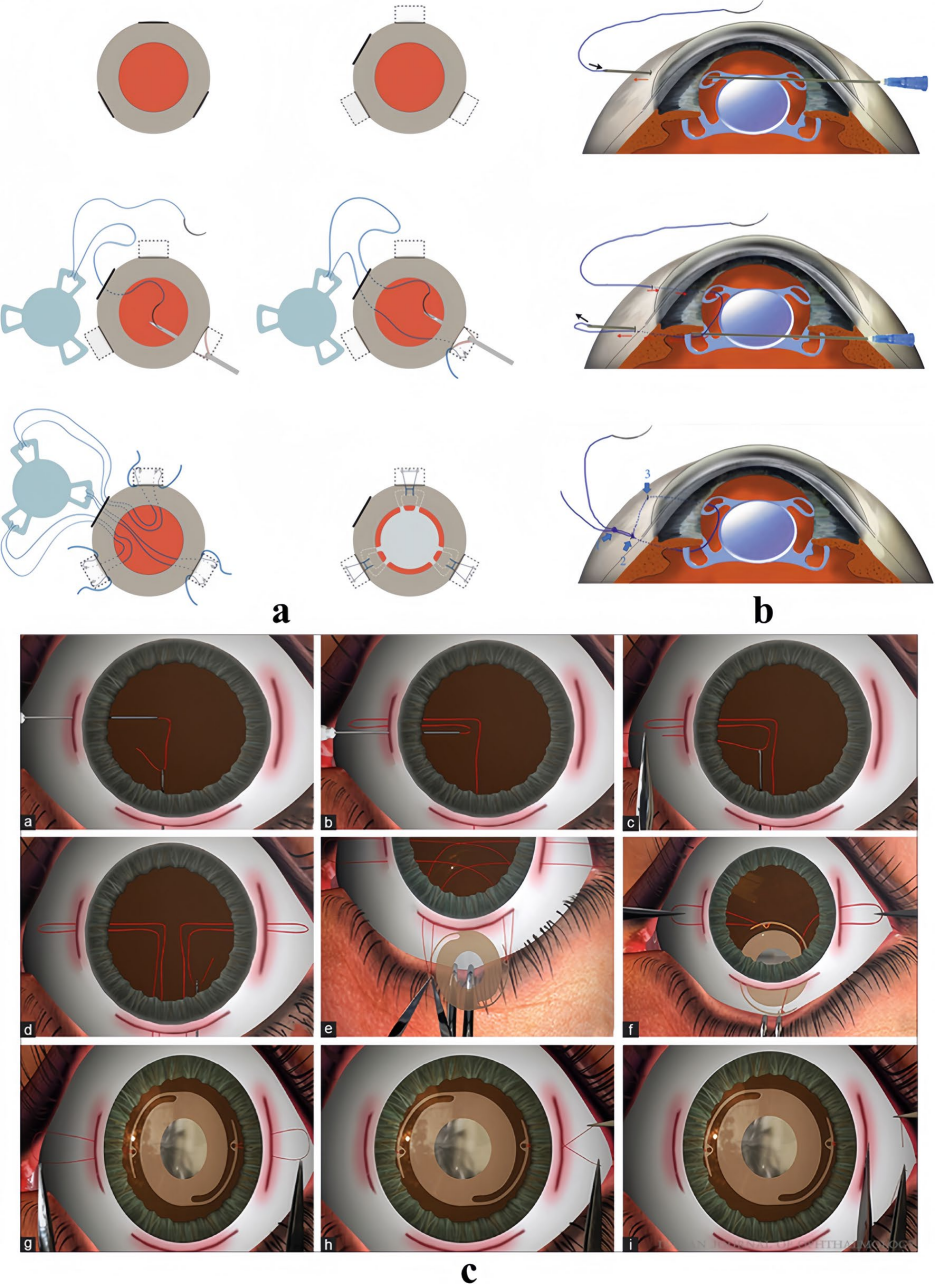
Diagrammatic representations of suture flapless scleral fixation. **a** Six-point transscleral suture fixation by making three scleral pockets. (Reproduced with permission from Ref. [51]). **b** Intraocular suture looping technique. The 8–0 polypropylene suture was put into the guiding needle and then passed through the first fixation site of the sclera, the first eyelet of the IOL haptic, then the second eyelet of the homolateral IOL haptic. The IOL is fixed by fixation and anchor knots. (Reproduced with permission from Ref. [58]). **c** Modified sewing machine technique. Main surgical procedures: creation of suture loops on both sides of the IOL eyelets, adjusting the IOL centration, tying the free ends of the suture, and pushing knot into the tunnel. (Reproduced with permission from Ref. [63])

In addition, a six-point transscleral suture fixation by making three scleral pockets has been recently reported (Fig. 1a) [51]. This method uses an 8–0 polypropylene suture to secure a 3-looped haptics one-piece IOL (CT ASPHINA 603P, Zeiss) [51]. This technique reduces the size of the scleral pocket and effectively reduces intraoperative bleeding and operation duration. Ni et al. conducted a study with a mean follow-up of 7.8 months, where 4 out of 21 patients (19%) with transient corneal edema, and 3 patients (14.3%) with transient increased IOP were observed on the first day after operation [51]. No complications, including suprachoroidal hemorrhage, vitreous hemorrhage (VH), retinal detachment, suture breakage, IOL tilting, or IOL decentration, were observed [51]. Yu et al. observed no other significant complications besides transient IOP elevation [39]. The theoretical basis of this technique is that the three pockets create a plane which facilitates more stable fixation and concentration [39]. Simultaneously, the three-looped haptics IOL (CT ASPHINA 603P, Zeiss, Germany) possesses a square-style frame accompanied by a wide foundation, significantly decreasing the probability of pupillary capture of IOL [39]. Ultimately, the scleral pocket ensures that the patient’s conjunctiva remains intact and devoid of scarring [39].

In summary, the technique of creating the Hoffman scleral pocket transscleral fixation is a relatively safe and practical method that has been utilized for different types of IOLs.

#### Z-suture technique

Since its inception in 2010, Z-suture technology has undergone extensive verification [17]. The physical basis of the Z-suture technique is that the angle at the Z-suture reentry can increase the friction force of the suture to a great extent; The anatomical basis is that the sclera is composed of dense, interlaced, irregularly arranged fibrous tissue, which also increases the friction on the suture.

Szurman and colleagues first described the Z-suture transscleral fixation technique for PCIOL (AF-1; Hoya, Frankfurt, Germany) using 10–0 polypropylene sutures [17]. The Z-suture was passed five times and then secured into the sclera [17]. These 67 eyes were observed with a mean follow-up time of 22.4 months. Except for 3 eyes with transient mild ciliary hemorrhage, no suture erosion, suture looseness, scleral atrophy or chronic inflammation occurred in any of these patients [17]. Dimopoulos et al. reported that the incidence of suture erosion or exposure in patients could be reduced to zero, and the mean corrected distance visual acuity was 0.73 ± 0.55 logarithm of the minimum angle of resolution (logMAR) at five years after surgery [52]. However, after further investigation, it was discovered that the occurrence of postoperative IOL (AcrySof^®^ MA60AC, Alcon) tilt was as high as 72.3% in patients who underwent this procedure [37]. The use of sutures with varying diameters, including 9–0, 8–0, and 7–0, has been documented [53–55]. John et al.’s study confirmed that scleral suture fixation of PCIOL using 8–0 polypropylene sutures can safely and effectively improve patient vision [46, 56].

In the Z-suture technique, certain operational considerations are crucial. Paracentesis at the 3 o’clock and 9 o’clock positions should be avoided due to the presence of the long posterior ciliary artery, which poses a significant risk of bleeding. Instead, the surgeon can opt for the 2 o’clock and 8 o’clock positions, away from the main incision site, for greater convenience during the procedure. It is advisable to insert the needle to a depth ranging from 1/3 to 1/2 of the scleral thickness. Insertion should not be too deep as it may result in inadvertent intraocular penetration, nor too shallow as it could lead to suture exposure and allow the entry of microorganisms and subsequent endophthalmitis. Precise positioning of the entry point is imperative to minimize complications. At each Z-suture, the insertion of the needle should align with the exit point of the preceding needle. Following the completion of the final stitch, it is essential to tightly secure and trim the suture close to the conjunctiva to prevent exposure, and thus avert the risk of endophthalmitis resulting from excessively inappropriate suture length.

The Z-suture fixation technique meets the necessary tension force for IOL fixation and prevents suture knot complications. Its safety has been clinically verified; however, its long-term stability requires further observation. In addition, the tension generated by bilateral loops with three stitches (Z-suture) is sufficient to stabilize the IOL (OSF 651, Optima Lens, Excellent Hicare Pvt Ltd, India) [57].

#### Intraocular suture looping technique

In simple terms, the technique is an intraocular suture looping assisted by two 27-gauge/30-gauge needles (Fig. 1b) [58]. The 8–0 polypropylene suture was put into the guiding needle and then passed through the first fixation site of the sclera, the first eyelet of the IOL (Akreos AO60, Bausch and Lomb, North Clearwater, FL) haptic, then the second eyelet of the homolateral IOL haptic, and finally, the second sclerotomy, carrying out the same set of manipulations on the opposite side [41]. After ensuring that the tension of the sutures on both sides was properly adjusted to align the IOL, the needle was bent from the first fixation site to the second fixation site [41]. The sutures’ ends were then tied with fixation knot (the first knot) and attached to the sclerotomy on both sides. Further, an anchor knot (the second knot) was created approximately 2–3 mm from the fixation knot [41]. The fixation and anchor knots were buried in the scleral tunnel, with the fixation knot utilized for IOL fixation and the anchor knot to guide the ends of sutures for burying in the tunnel [41]. The mean postoperative follow-up was 5–15 months, during which the IOL remained in a stable position and the visual outcome significantly improved in all 11 eyes after surgery. No evidence of suture loosening, suture erosion, scleral atrophy, chronic inflammation, retinal tear, or detachment was identified during the follow-up period [41].

This approach incorporates the essential advantages of the friction knot technique in preventing suture erosion and streamlines the steps required for repositioning a dislocated IOL or IOL-capsular bag complex while guaranteeing surgical effectiveness and safety. The approach uses 8–0 polypropylene suture, which is thicker and boasts higher tensile strength than 10–0 polypropylene suture, thereby improving intraoperative manipulations and reducing the risk of late IOL dislocation owing to suture breakage [59]. However, further assessment is necessary as the occurrence of dislocations of the IOL with four haptics administered intraoperatively is relatively low [60, 61]. The examination should encompass additional cases with various types of IOL haptics and other potential complications.

#### Sewing machine technique

The sewing machine technique, also known as the Cobbler’s technique, was originally invented by Indian physicians [62]. This method entails the creation of broad scleral flaps or grooves to enclose the sutures and protect them from the scleral surface [63]. The principle of sewing machine technology is to complete the suture technology of two tissues with a single suture [63]. This technique was initially employed for repairing iridodialysis or ciliary body detachment [62]. Follow-up operators have adapted the sewing machine technique for IOL fixation (Fig. 1c) [63].

The technique involves creating suture loops on both sides, tying IOL haptics at both ends of the free suture, and implanting the IOL through the main incision. The free end of the suture is knotted and pushed into the scleral tunnel. In addition, a modified technique that uses 8–0 polypropylene suture for the repair of traumatic cyclodialysis clefts is also available [64]. The primary benefit of the sewing machine technique is its ability to sidestep the necessity of forming the scleral flap or groove, and the operation is more minimally invasive [64]. Even in the state of severe scarring of the conjunctiva, the operation can be completed without clipping the conjunctiva [64]. This technique can also be extended to other clinical situations except for iris coloboma repair and IOL fixation such as Cionni ring fixation and repair of subluxated IOL [63, 64].

#### Cow-hitch knot

Mantopoulos et al. developed a novel approach for fixation PCIOL (CZ70BD IOL, Alcon Laboratories, Inc, Fort Worth, TX) with Gore-Tex sutures using a cow-hitch knot [65]. The cow-hitch knot is created with the externalized suture and secured by tensioning the knot over the corresponding islet of the IOL haptic [65]. This innovative fixation method uses a Gore-Tex suture with high tensile strength, which can effectively reduce the risk of perioperative and even long-term postoperative suture degradation, and increase the possibility of permanent IOL fixation [65]. At the same time, the suture fixation for PCIOL at four points of the sclera through a very simple cow-hitch knot ensures the neutrality and stability of the IOL as well as reduce the risk of IOL tilt [66].

Bonnell et al. carried out a retrospective chart review of this technique involving 14 patients who underwent fixation of PCIOL (CZ70BD IOL, Alcon Laboratories, Inc, Fort Worth, TX) with a cow-hitch knot over 14 months [66]. After treatment of aphakia eye or IOL dislocation, visual acuity improved significantly in all patients. In terms of the procedure’s safety, short-term complications linked to IOP (40%, associated with steroid use after surgery), CME (27%), mild VH (20%, without additional intervention), and corneal edema (13%) may occur during the 1-year postoperative follow-up period [66]. Long-term complications such as iris capture of IOL (14%), suture exposure (7%), macular pucker (7%), optic neuropathy (7%), and retinal detachment (7%) were also reported [66]. Unsal et al. evaluated the surgical and refractive outcomes of this technique utilized in the management of subluxated IOLs [67, 68]. The analysis incorporated 19 eyes with a mean follow-up of 10 months (range 6–15 months) [68]. The risk of VH and elevation IOP was 5.26% and 10.52%, respectively [68]. No instances of IOL tilting or dislocation, conjunctival erosion, or macular cystoid edema were observed during the follow-up period [68]. A method employing twin-armed single sutures was also noted as a possible approach to tying the cow-hitch knot [34]. Of the patient cases studied postoperatively, 8 cases experienced transient IOP abnormalities, 2 cases had IOL pupillary capture, and 1 case exhibited suture exposure [34].

In conclusion, suture scleral fixation of IOL via a cow-hitch knot with Gore-Tex sutures offers distinct advantages for secondary IOL fixation in patients who lack capsular bag support, especially after ocular trauma. While some patients may experience short or long-term postoperative complications, relatively speaking, no serious long-term complications related to the IOL were observed.

#### Adjustable buckle-slide suture

Symmetrical and balanced tension of sutures is crucial for fixing the IOL (Akreos AO60, Bausch & Lomb, North Clearwater, FL) in a well-centered position in transscleral suture fixation [56]. Conventional suturing techniques necessitate readjustment of suture tension during fastening to the sclera [56]. Once the suture is attached to the sclera, the tension cannot be adjusted to optimize the position of the IOL [56]. Zhao et al. presented a novel surgical technique for transscleral suture fixation by creating an adjustable and less traumatic buckle-slide device [56].

A buckle-slide device typically comprises of a frame with grooves that allow straps to be passed through, much like the adjustment device for school bags in daily life [56]. After the IOL is implanted into the eye with a conventional four-point fixation technique, an additional suture (a sliding line for adjustment) is used at the end of each suture to create the buckle-slide device [56]. The study enrolled 12 patients with an average age of 56 years (range 19 –73 years) and a mean follow-up period of 9.3 ± 8.5 months (range 3–16 months) [56]. The visual acuity preoperatively was 0.80 ± 0.72 logMAR, while 0.29 ± 0.42 logMAR at the final follow-up. Intraoperative complications were not observed [56]. Postoperative complications included transient hypertension (1/12) and transient VH (1/12) [56]. No hypotony, scleral atrophy, retinal detachment, or suture-related complications were observed [56]. The IOLs remained centered throughout the follow-up period [56].

The major advantage of the buckle-slide structure lies in its ability to provide adequate friction for scleral fixation paired with convenient adjustability [56]. As compared to the flap technique, there is a lower risk of scleral dehiscence and suture erosion. Additionally, it has the added benefit of less trauma, minimal conjunctival openings, and reduced manipulation of the sclera, making it a suitable option for individuals with glaucoma or those who are at risk of glaucoma surgery [56]. The buckle-slide device is a highly versatile tool for IOL fixation, capable of application irrespective of the suturing technique, material [polypropylene or polytetrafluoroethylene (PTFE)], or design (straight or curved needle)[56]. However, due to the complexity of the operation, it is more time-consuming in cases where repeated operations are required to create the buckle-slide device (such as the four-point or six-point fixation technique) [56]. Additionally, many suture ends are exposed after the buckle-slide is knotted, and it is crucial to carefully trim these ends once the tension has been adjusted [56]. Nevertheless, it seems that there are no more studies to verify the long-term stability of this device, and it should also be compared with other fixation methods.

#### Thread winding technique

To prevent decentration or tilting of the IOL caused by suture erosion or breakage in the suture fixation technique, Lee et al. devised a novel method for transscleral fixation of the PCIOL (Alcon, Fort Worth, TX, USA) [69]. Instead of suturing the polypropylene thread to the IOL haptics, the thread is wound around the haptics, the ends are then sutured, and the conjunctiva covered with fibrin glue [69].

Surgical outcomes and postoperative complications by comparing them with the conventional transscleral fixation method was studied [69]. Nonetheless, no significant difference in postoperative best-corrected visual acuity (*P* = 0.13) and mean sphere (*P* = 0.42) was discovered between both groups. The thread winding group demonstrated a significantly lower mean cylinder than the conventional group (*P* = 0.01) [69]. Long-term follow-up (> 18 months) of 15 patients using this surgical technique revealed no complications except for temporary IOP elevation (2/15, 13%) [69]. However, complications such as IOL decentration (8/48, 17%) and CME (4/48, 8%) occurred in the conventional fixation group [69]. Therefore, the thread winding technique is favored because of its simplicity, mechanical stability, avoidance of suture-related complications, shorter mean surgical time (*P* = 0.0001), and is better than previously reported techniques [69]. Subsequently, Yoon et al. improved this technology and compared it with the modified Yamane technique [70]. They demonstrated that the IOL (AMO Sensar AR40e, Johnson & Johnson Vision) was located at 2.5 mm posterior to the limbus, and the postoperative refractive prediction error was minimal [70]. The IOL implanted with the modified Yamane technique is positioned further back than the IOL implanted with the thread winding technique, which is closer to the 2.5 mm posterior to the limbus [70]. However, there was no significant difference in postoperative vision and astigmatism, meaning that both surgical techniques were effective in terms of IOL stability and good postoperative refractive outcomes [70].

It is worth noting that at present, there are no other relevant reports on this technique. The existing reports also have limitations such as small sample size, non-random allocation of patients, and its unknown safety profile [69].

#### Flapless one-knot suture

The postoperative optical performance and clinical outcomes are significantly influenced by various factors, including IOL design, in addition to surgery techniques. Depending on the haptic design, IOLs can be classified as either plate-haptic IOLs or open-loop such as J-loop, L-loop, and C-loop IOLs [71]. Open-loop IOLs may perform better than plate-haptic IOLs [72–74]. As a result, a novel suturing method for transscleral fixation of C-loop IOLs (Tecnis ZCB00, Johnson and Johnson vision, USA) has been reported [75]. Only single-knot is necessary, reducing the possibility of scleral stimulation and endophthalmitis when compared to other techniques that involve more knots [75].

During the surgical procedure, two lateral incisions were made from the corneal limbus at 2–4 and 8–10 o’clock [75]. The main procedure is to pass a 10–0 polypropylene suture through the 8 o’clock position, the main incision (through the haptic of the IOL), the 4 o’clock position, the 2 o’clock position, the main incision (through the other haptic of the IOL), and the 10 o’clock position [75]. The suture was adjusted and tightened to attain the centration of the IOL. Afterward, a secure knot was tied and buried in the sclera. Post-operatively, visual acuity improved in all 16 eyes [75]. No optic capture, suture exposure, IOL dislocation or decentration, or CME was observed [75].

The research had a relatively limited follow-up duration, averaging 17.2 ± 3.9 months, which implies that late suture-related complications may have gone unreported. Moreover, the sample size was limited [75]. It is imperative to conduct additional research studies that encompass larger samples, a more comprehensive collection of data, and a longer follow-up duration to ascertain the clinical efficacy and outcomes. Nevertheless, the technique’s simplicity, reliability, and stability remain undeniable [52].

## Sutureless scleral fixation of IOL

In some special cases, suture fixation is unsuitable, such as a non-constricting pupil, ischemic retinopathies, peripheral anterior synechia, and large sectoral iridectomy [76, 77]. Modified suture fixation, however, still necessitates the use of sutures and hence does not entirely prevent the complications that arise from suture breakage. Most techniques for making scleral flaps/pockets are time-consuming and intrusive. A novel surgical procedure, intrascleral sutureless fixation of IOL, has been created to achieve successful fixation of the IOL and avoid suture-related complications. The crucial step during the surgery is to insert the IOL haptic into the needle, and fix the IOL haptic between the intrascleral tunnel, and accurately position the IOL so that the position of the IOL after the operation is centered to avoid the glare and astigmatism caused by the IOL tilt.

Sutureless scleral fixation of IOL was initially reported by Maggi and Maggi in 1997. The IOL (Teflon^®^) haptics used in this technique are made of 8.5 mm long PTFE [78]. Subsequently, Gabor et al. proposed the sutureless intrascleral three-piece PCIOL fixation in 2007 [26]. In 2008, Agarwal et al. made an important addition to this procedure with the use of fibrin glue-assisted intrascleral IOL fixation [32]. These advancements demonstrate that intrascleral sutureless IOL fixation is an effective, safe, and practical surgical method. The use of compressed haptic tips, highlighted in the study by Ucar et al., enhances haptic stabilization, making this technique increasingly preferred in clinical practice. Flattened haptic tips, particularly in pediatric cases, provide secure fixation without the need for sutures or glue, reducing the risk of complications and improving postoperative outcomes.

### Gabor’s technique

Gabor’s technique is employed for subluxated and luxated cataract and secondary IOL implantation [26]. This method involves a straightforward procedure for standard three-piece IOL fixation without the need for specific haptic design or preparation [26].

During the surgery, two symmetrical scleral tunnels, 180° apart, are created parallel to the corneal limbus, each measuring approximately 2 to 3 mm in length and located 1.5 to 2.0 mm from the corneal limbus to a depth of around 1/2 scleral thickness [26]. The sclera is punctured using a 24-gauge cannula [26]. The haptic on one side of the IOL is pulled out of the scleral puncture site using 25-gauge end-gripping forceps and fixed in the scleral tunnel [26]. This process is repeated for the opposite haptic, and by accurately positioning the IOL haptics within the scleral tunnel, the axial IOL position is stabilized, minimizing IOL tilting and dislocation [26]. Since the haptic is buried, there is no occurrence of conjunctival erosion. Chronic inflammation and hemorrhage risks are lower with this technique compared to suture fixation methods. It is suitable for standard three-piece IOLs but not for the more recent one-piece acrylic or silicone IOLs [26].

**Fig. 2:**
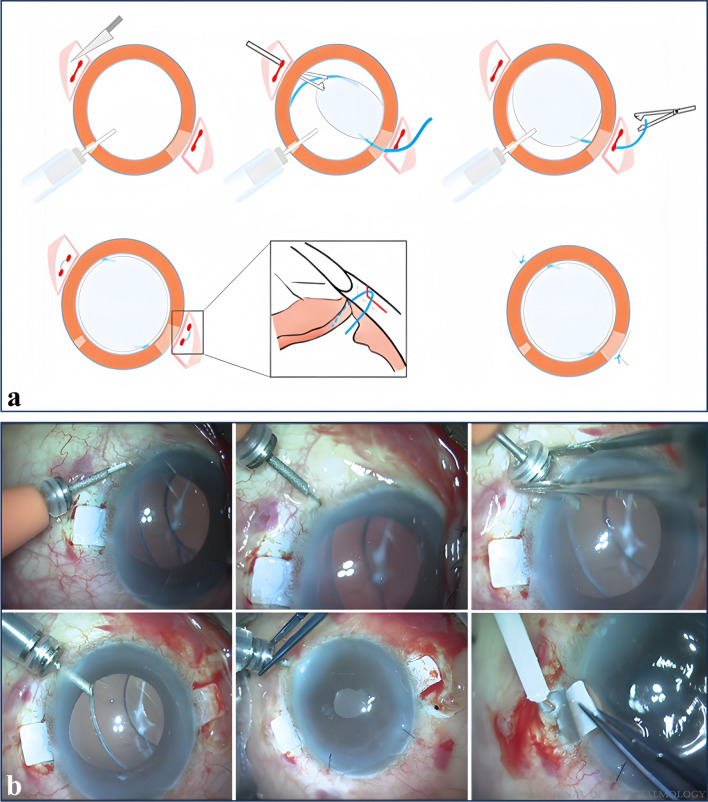
Agarwal’s glued IOL technique. Fibrin glue is applied, and the flap is sealed down over the haptic. (Reproduced with permission from Ref. [98])

Performing this technique can be challenging due to the intraocular grasping of the IOL haptics and fixation in the scleral tunnel, potentially increasing intraocular complications. Additionally, creating the scleral tunnel requires proficiency as the IOL is susceptible to deviation. Leakage from the incision may result in intraocular infections [26].

### Agarwal’s glued IOL technique

Agarwal has described a novel surgical method for implanting PCIOL in eyes with defects or absence in the posterior capsule by using biological glue (Fig. 2) [32]. This technique successfully attained good flap closure and IOL stability and centration, preventing suture-related complications [32].

During the operation, two identical 180-degree scleral flaps were created at a distance of 1.5 mm from the corneal limbus [32]. These flaps measured approximately 2.5 to 3.0 mm in size [32]. Next, the sclera was punctured using a 22-gauge needle, and the IOL haptic was maneuvered out of the puncture site using an endgripping 25-gauge micro-incision capsulorhexis forceps beneath the scleral flap [32]. Finally, fibrin glue is injected under the scleral flap, and pressure is applied for 10 to 20 s [32]. The technique’s primary purpose is to place the IOL haptics beneath the scleral flap. If the haptic is excessively long, the scleral flap must be enlarged to accommodate it. Furthermore, fibrin glue-assisted fixation must ensure that the adhesive area is completely dry. Otherwise, the incision is prone to leaking, resulting in intraocular infection. Ensuring the flap remains dry is crucial, however, puncturing the sclera with a thick needle, such as a 22-gauge, can create a large opening that leads to continuous fluid leakage from the eye. This constant leakage makes it challenging to keep the area under the flap dry.

Agarwal’s study did not encounter any significant complications during the 6-week follow-up period [32]. Various studies have used fibrin glue for sutureless scleral fixation and reported postoperative IOL decentration of 1.9% to 6.9%, and 1.4% to 7.9% CME [79–82]. However, other postoperative complications varied significantly based on sample size and follow-up time. For instance, Kumar et al.’s study only mentioned intraoperative hemorrhage (3.7%), IOL decentration (5.6%), and CME (7.5%) as complications [79]. However, other studies have reported additional complications, including VH, anterior uveitis, chronic vitreitis, and IOL haptic displacement [80].

This technique minimizes the occurrence of complications related to sutures and seals the scleral incision, and thus prevents the onset of postoperative hypotony or endophthalmitis due to incisional leakage. Nevertheless, the procedure faces challenges in widespread applicability due to the high cost of fibrin glue and difficulty in procuring it.

### Yamane technique

The procedure developed by Gabor and Agarwal is complex, requiring extensive conjunctiva incision or scleral flap creation, and the puncture is prone to leakage. Subsequently, several modified procedures have been derived. Despite this, some scholars have noted that simple intrascleral sutureless fixation is unstable and cannot fully maintain IOL centering [83–85]. Thus, Yamane and co-workers further improved the sutureless intrascleral fixation technique [27].

The Yamane technique aims to pull the haptics of the three-piece IOL [X-70 (Santen, Osaka, Japan]; PN6A (Kowa, Tokyo, Japan); Tecnis ZA9003 (Abbott Medical Optics, Santa Ana, CA); or MA60MA (Alcon Laboratories, Inc)] through two sclerotomies using a 30-/27-gauge needle [27]. The haptics of the IOL are inserted into the needle cavity with the forceps. Then, the haptics are inserted out to the surface of the conjunctiva with the needle [27]. The end of the cryogenic cauterized haptics was used to make a flange and embedded in the scleral tunnel to provide sufficient friction to maintain the centration of the IOL optic [27]. The flange effectively prevents the dislodgement of haptics back into the posterior chamber, ensuring the successful and secure placement of the three-piece IOL in the absence of posterior capsule support [27]. Compared with previous surgical techniques, the Yamane technique offers a simpler approach while providing more reliable IOL fixation. In a study of 97 patients (100 eyes) with a mean follow-up of 20.6 months, no instances of IOL dislocation or significant IOL tilt were observed [27]. Postoperative complications mainly included iris capture (8%), VH (5%), and CME (1%) [27]. If the IOL is not properly centered or is tilted, either the angle of insertion of the two fixation positions is not 180°, the two sites may have been inserted at different distances from the corneal limbus, or the scleral tunnels may have varying lengths and/or orientations [86]. As for the tunnel creation, it should be created approximately parallel to the corneal limbus, with a slight backward angle of about 5° from the limbus, but no more than that. It may be feasible to increase the angle of the tunnel slightly beyond 5° for myopia patients or decrease it for hyperopia patients. This is because the bigger the eye, the shorter the available haptic length will be.

Although any three-piece IOL can be used in the Yamane technique, IOLs with polyvinylidene fluoride (PVDF) haptics are preferred choices because of their durability and ability to avoid kinks or breaks; most surgeons recommend the CT Lucia 602 IOL for this procedure [87]. The Yamane technique has become increasingly popular due to its many benefits, including no need for sutures, glue, scleral or conjunctiva stripping, minimal surgical trauma, good IOL stability, and a relatively short operative time [88]. Additionally, controlling the puncture location and the length of the tunnel is crucial for this technique.

Despite the benefits of the flange, concerns remain regarding its ability to provide adequate friction within the scleral tunnel due to its round geometry. Enhancements in haptic stabilization, particularly for cases with more flexible sclera such as pediatric aphakia, are necessary to ensure stable fixation. Additional methods to increase friction and stabilization within the scleral tunnel should be considered for improved outcomes.

### Flattened flanged fixation technique

**Fig. 3:**
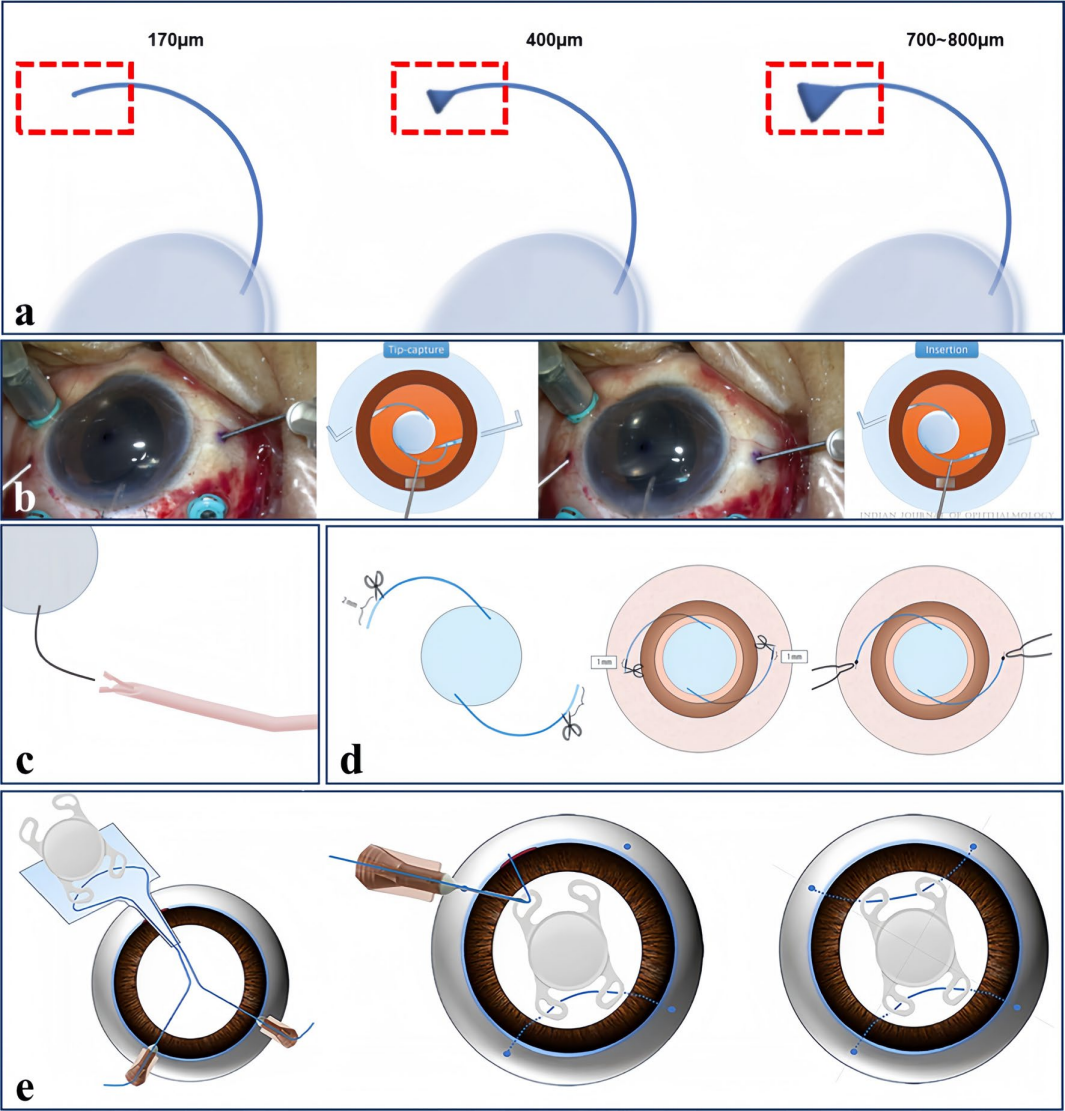
Diagrammatic representations of modified Yamane techniques. **a** Flattened flanged intrascleral fixation technique. The IOL haptic (dashed box) had a diameter of 170 μm; the diameter of the flange was 400 μm; the diameter of the flattened flange was 700 to 800 μm. **b** Modified Yamane technique with 26-gauge needles. (Reproduced with permission from Ref. [166]). **c** Operation of the forceps-needle device. **d** The double trimming of the haptics. The 2.0-mm section was cut off from the haptics before the IOL was inserted into the anterior chamber, and a 1.0-mm section was cut off the haptics after the implantation. (Reproduced with permission from Ref. [102]). **e** Four-flanged technique. Two flanges outside and two flanges in the eyelets of the IOL. (Reproduced with permission from Ref. [167]

The improvement of this technique is that the tips of the haptic on both sides of the IOL (Sensar AR40, Abbott Medical Optics, Santa Ana, California, USA) are cauterized into flanges and then held with a needle holder to make them flat and increase the surface area in contact with the intraocular tissue [89]. Consequently, this technique increases the diameter of the flange from 400 μm to 700 μm (IOL haptics 170 μm), allowing it to be fixed more firmly in the scleral tunnel with a diameter of 360 μm (Fig. 3a) [89]. In their study, there was no IOL dislocation (0%) in the flattened flanged group (*P* < 0.32) and postoperative IOL dislocation in 1 eye (2.5%) in the original Yamane technique group [89]. In another study that explored the clinical outcomes of this technique in children (2–15 years old), the flattened flange (Sensar AR40, Abbott Medical Optics, Santa Ana, CA) tip was up to 800 μm in diameter [90]. The outcomes show that the flattened flanged fixation technique can be applied to cases of pediatric aphakia with good visual effects, minimal side effects and stable IOL [90].

Further study is required to obtain long-term outcomes. What is clear is that the flattened flanged fixation technique can provide superior IOL stability and less tilt compared to the Yamane technique [89–92].

The application steps of the technique include some simplified steps compared to the Yamane technique. When the IOL is in the injector, the first haptic is pushed into a 27-gauge needle to extract it outside the sclera. Once the haptic is extracted outside the sclera and conjunctiva, the tip is cauterized to form a flange. After cauterization, the haptic tip is flattened with a needle holder to prevent the first haptic from being removed from the sclera during the manipulation of the second haptic. The flattened and expanded haptic tip is then pushed into the enlarged outer edge of the scleral tunnel due to needle traction, ensuring it is secured within the scleral tunnel. This process prevents unnecessary stress for the surgeon during the second haptic manipulation. The surgeon repeats the same cauterization and flattening process for the second haptic [89, 90]. To facilitate the manipulation of the second haptic, the edge of the IOL optic is pushed towards the opposite scleral tunnel with the needle. Thus, along with the IOL optic, the trailing haptic also approaches from the periphery to the center, making it easier to reach the trailing haptic with intraocular forceps and ensuring the haptic tip is held in a more appropriate position. The approach of the trailing haptic from the periphery to the center and the placement of the straightened distal part of the haptic towards the direction of the needle facilitate relatively easy and gentle placement of the haptic into the lumen with a single use of forceps [91].

### Canabrava’s technique

A novel technique for sutureless intrascleral fixation of a single-piece nonfoldable IOL has been formulated; this builds on Yamane’s double-needle technique [93].

This technique employs a 5–0 polypropylene suture passed through the IOL eyelet. The suture’s ends are cauterized and expanded to form two flanges in the eyelets and two flanges outside to secure IOL (OP-72; Mediphacos, Belo Horizonte, Brazil) inside the scleral tunnel [93]. There were no postoperative complications as confirmed at the 4-month follow-up [93]. Some experts have adapted this procedure to repair wide iridodialysis [94]. Other modifications include the use of a 6–0 PROLENE suture for scleral fixation [95]. Postoperative complications have been reported, including one case of exposure of the suture bulb through the conjunctiva and two cases of suture slippage from the haptic [95]. The slippage may have been caused by the small flange size along with the haptic being stuck and the plunger of the injector during IOL insertion [95]. The revised method necessitated a diminutive corneal incision and a shorter learning curve, and the usage of McPherson’s forceps facilitated the threading procedure, as compared to Canabrava et al.’s original technique [95]. It is important to note that this technique also marked the first instance of employing 6–0 PROLENE sutures for the single-piece foldable IOL fixation [95].

This novel method enhances, streamlines, and expedites the intrascleral fixation of the single-piece nonfoldable/foldable IOL. Additionally, it successfully resolves the difficult procedure for haptic engagement and externalization in the Yamane technique, all without necessitating a specific 30-gauge thin-wall needle.

## Modified Yamane technique

To enhance the simplicity and safety of the Yamane technique, several modifications have been developed. However, further observation of clinical outcomes is still necessary.

### 26-gauge needle

Yamane’s original surgical procedure necessitated a specially designed 30-gauge thin-wall needle. Nevertheless, the 26-gauge needle, which has a 0.25 mm inner diameter, additionally has adequate space to accommodate an AR40e IOL (Tecnis AR40e, Abbott Medical Optics) haptic, measuring 0.15 mm in diameter [96]. Hence, an improved technique using 26-gauge needles was reported [96, 166].

The Yamane technique initially necessitated a minimum of two corneal incisions at the superonasal and superotemporal quadrant [27]. The haptic was then introduced into the puncture needle cavity using intraocular forceps [27]. Improper grasp of the IOL haptics during needle insertion might result in the kink or breakage of haptics, which leads to IOL tilt or decentration, or even the replacement of a new IOL [96]. The technique has since been modified to use a single corneal or corneoscleral incision at 12 o’clock. The scleral incision is made at the 3 o’clock position, 2.0 mm from the corneal limbus, using a 26-gauge needle through the conjunctiva (Fig. 3b) [96]. About half the length of the haptic is inserted into the 26-gauge needle, with the remaining trailing haptic outside the main port to avert IOL displacement into the vitreous cavity [96]. A second sclerotomy was conducted at the 9 o’clock using a 26-gauge needle [96]. The trailing haptic is introduced through the corneal incision at the 12 o’clock position and placed into the cavity of the second needle with the help of intraocular forceps [96]. The direction of the trailing haptic end is aligned parallel to the needle tip opening, resulting in simplified haptic insertion [96].

This technique stresses leaving half the haptic’s length in the puncture needle before externalization, which offers a simpler and safer approach to inserting the haptic into the needle tip. This also decreases the probability of bending or breaking the IOL haptic. Importantly, the slightly larger inner diameter of the 26-gauge needle helps minimize vitreous traction during scleral penetration, thereby reducing the risk of retinal detachment. Additionally, the 26-gauge needle can accommodate larger sutures, facilitating the creation of scleral tunnels more smoothly and reducing the risk of postoperative IOL dislocation or rotation, thereby enhancing fixation stability.

### 27-gauge trocar

Intrascleral sutureless fixation of IOL utilizing 27-gauge needles necessitates six surgical wounds. Ishikawa et al. sought to lessen the number of surgical wounds to four via the use of two 27-gauge trocars for vitrectomy and IOL (NX‐70; Santen, Osaka, Japan) fixation [97].

The modified technique was applied to three 27-gauge trocars, two of which were set 2 mm behind the limbus at 2 and 8 o’clock, and the double-needle technique was used to fixate the IOL haptics [97]. Once the IOL haptic was guided in, the trocar and the needle were withdrawn simultaneously with the haptic’s end being cauterized into a flange [97]. As the scleral tunnel formed by the 27-gauge trocar was larger than that created by the 27-gauge needle, irrigation length of 1.5 mm for IOL haptics was necessary within the 27-gauge trocar group to prevent IOL dislocation during and after the procedure [97]. This was compared to 0.7 mm in the 27-gauge needle group [97]. No significant differences were observed between the groups in terms of changes in visual acuity, corneal endothelial cell counts, refractive error, astigmatism, or surgery-related complications [97]. The study findings revealed that phacoemulsification was the most prevalent factor in IOL fixation, which indicates a likelihood of glaucoma surgery in future patients undergoing the procedure [97]. Therefore, the adapted technique minimizes the surgical wounds, ensuring that the integrity of the conjunctiva is preserved [97].

The refined technique is similarly effective to the original but entails less invasiveness. Therefore, flanged fixation using the double-needle technique via a 27-gauge trocar needle is a favorable technique for IOL fixation [97].

### Haptic-twist method

The Yamane technique presents a challenge in securing the trailing haptic, as the trailing haptic becomes immobile and sometimes invisible once the leading haptic is fixed in the needle cavity [88]. This can pose difficulty in the trailing haptic entering the needle cavity, and misalignment of the haptics and needle may result in the operator performing aggressive manipulations, leading to deformation or even breakage of the haptics [84, 98]. To address these challenges, a novel technical advancement involves twisting the IOL (AcrySof MA60AC, Alcon Laboratories, Inc) haptics during docking [88].

To execute this technique, the left hand grasping the intraocular forceps must be positioned dorsally with the hand held up, allowing the forceps to rotate before entering the eye [88]. The IOL haptic should be held approximately 2–3 mm away from its tip, and the forceps should be rotated 180° counter-clockwise around its own axis to give the trailing haptic a slight twist without deformation, aligning it to an appropriate angle to the needle cavity [88]. Subsequently, the IOL haptic should be extracted and cauterized to form a flange [88].

The material and design of haptics can affect the difficulty of the procedure [72, 99]. Currently, four materials are available: PMMA, polyimide, polypropylene (PROLENE), and PVDF [88]. Among these materials, PVDF is the most ductile and fracture-resistant. In this study, a rigid material, AcrySof MA60AC with PMMA haptics, was used [88]. Thus far, there have been no observed instances of the haptic bending or breaking during surgery [88]. As a result, it is believed that alternative materials possess sufficient durability to undergo this fundamental rotational maneuver [88]. Furthermore, no IOL optical tilting or decentralization occurred subsequent to the operation [88]. Observable complications were significantly milder compared to the original Yamane technique. Six eyes experienced moderate subconjunctival hemorrhage, while 2 eyes experienced mild VH, both of which were resorbed spontaneously during the procedure without any intervention [88].

These findings exhibit the versatility and efficacy of the innovative haptic-twist technique, along with its favorable surgical outcomes.

### Needle stabilizer

As the IOL haptics are secured in the scleral tunnel, it is crucial to consider the insertion angle of the thin-walled 30-gauge needle that is used to create the tunnel. Improper positioning of the needle insertion points or angles can lead to IOL tilting or decentration. To mitigate this issue, Yamane et al. devised an additional tool to ensure that the needles are properly inserted at the correct position and angle [100].

The needle stabilizer is handheld and has grooves on opposite sides of its ring-shaped body [100]. To create a scleral tunnel, the instrument should be used with the inner ring aligned with the corneal limbus and checked for horizontal placement [100]. Then, a 30-gauge needle is inserted at a 20° angle to the cornea and 10° to the iris surface to create the tunnels [100]. The needle stabilizer’s bottom surface has claws that grip the eyeball to keep the eye still during needle insertion [100]. The study compared postoperative outcomes in 25 eyes with needle stabilizers and 20 eyes without needle stabilizers [100]. The IOL tilt was 2.5° ± 2.5° in the group using needle stabilizers and 3.5° ± 3.1° in the other group [100]. Consequently, the application of the needle stabilizer significantly decreased IOL tilt (*P* = 0.02) [100].

The puncture fixator guarantees a steady and uniform insertion angle for the needle to sustain eyeball fixation and enhance the efficacy of IOL fixation using the Yamane technique. This instrument may assist surgeons with less experience [100].

### Forceps-needle

The intrascleral sutureless fixation techniques previously described may be effective in many cases. However, surgeons may encounter difficulties when threading the haptics through the needle despite repeated attempts [101]. This may be due to choosing an inner needle diameter that is too small relative to the IOL size (Acrysof MA60AT, Alcon Laboratories, Inc., or TECNIS CL Z9002, Johnson & Johnson Vision Care, Inc) haptics or an insufficient working angle between the end of the haptic and the needle [101]. Furthermore, the irregularities on the inner needle wall can impede the haptic’s entry into the lumen [101]. Additionally, the inadequate grip can cause the haptics to slide out from the needle lumen during the externalization maneuver [101].

To address the deficiencies of the existing methods, a “forceps-needle” has been created which amalgamates the traits of a forceps and a needle [101]. “Forceps-needle” means that a forceps is inserted inside the trocar, which can simultaneously satisfy a series of operations such as scleral puncture, IOL haptic seizing, and externalization (Fig. 3c) [101]. Subsequently, the tip of the IOL haptic is externalized until it forms a flange and is then embedded in the scleral tunnel [101]. The single-use forceps-needle device is available in two sizes: 27-gauge, with a inner lumen diameter of 0.3 mm and needle length of 10 mm; and 30-gauge, with an inner lumen diameter of 0.2 mm and needle length of 7.0 mm [101]. No complications were reported in any of the 10 patients included in the study [101].

This method simplifies the process, aiding intraocular maneuvers in both anterior and posterior segments [101]. Moreover, it enhances surgical safety [101]. The indications extend beyond primary aphakia and apply to IOL replacement and refixation in dislocated IOLs [101]. However, due to its novelty, utilization of the instrument for different techniques is limited, and further prospective studies are required to prove its added advantages.

### Trimming the haptic

When using the Yamane technique, untrimmed IOL (AR40e, Advanced Medical Optics, Santa Ana, USA) haptics may sometimes be excessively long because of variances in model lengths of haptics and individual differences [102]. Mere pushing of the haptics back into the scleral tunnel results in tension on the haptics that is not natural, and the consequent reaction force pushes the haptics out, causing the extrusion of the haptics [102]. Therefore, a study proposed trimming IOL haptics to an appropriate length during surgery and compared the long-term IOL stability and visual acuity outcomes between trimmed and non-trimmed IOLs [102].

In this technique, the haptic was trimmed twice [102]. During the first trim, both ends were trimmed by 2 mm to facilitate intraocular manipulation (Fig. 3d) [102]. The second trim was performed after adjusting the IOL, with an additional 0.5 to 1.0 mm trimmed as required to attain the optimal length (fully stretched while avoiding extrusion) (Fig. 3d) [102]. The haptic ends were subsequently cauterized to form an expanded flange and buried in the scleral tunnel [102]. The results indicate that there was no significant difference in best-corrected visual acuity, corneal endothelial cell density, IOL tilt at 3 months and 24 months post-surgery, and IOL decentration at 3 months post-surgery between the two groups of patients (*P* > 0.05) [102]. However, a statistically significant difference (*P* < 0.05) was observed when comparing the IOL decentration at 24 months post-surgery between the two groups [102]. IOL tilt and decentration saw an increase in both groups at 24 months post-surgery, in contrast to 3 months post-surgery (*P* < 0.01) [102].

To summarize, while the position of the IOL may alter over time, modifying the haptics to the optimal length enhances the IOL’s stability. While trimming the haptics can enhance IOL stability, it is debatable whether this method is effective in every case. Trimming the haptics can provide long-term stability but it may not yield the same positive results for every patient.

### Modified flanged fixation

#### The laser and spirit lamp

Besides the use of disposable thermal cautery, alternative techniques are available for creating a flange.

**Fig. 4:**
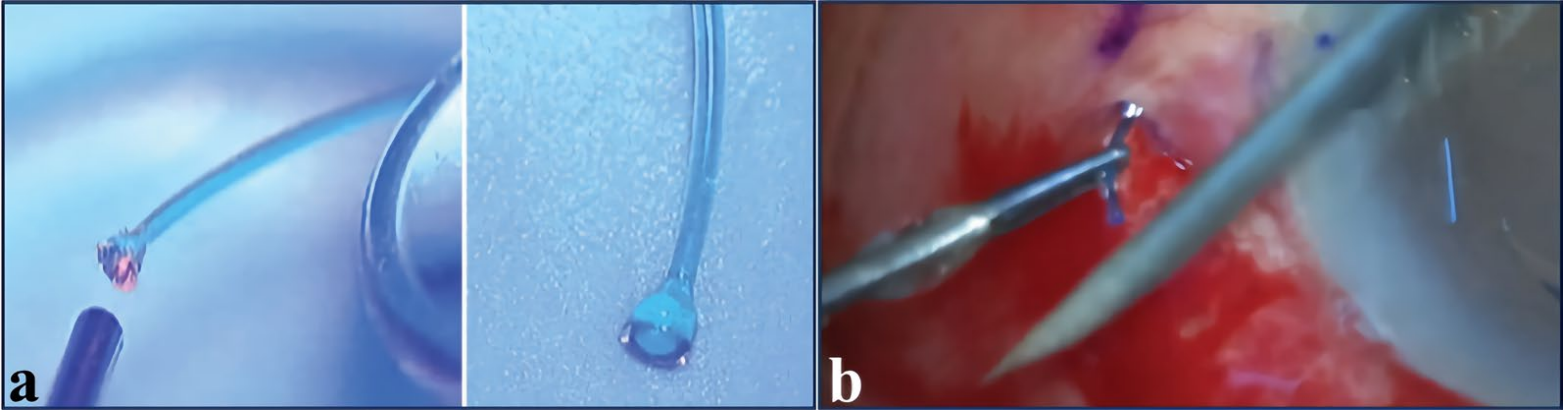
Intraoperative photos of modified Yamane techniques. **a** Creating the flange with a 532-nm Laser. (Reproduced with permission from Ref. [103]). **b** Creating the flange with the heated tip of a 26-gauge needle. (Reproduced with permission from Ref. [103])

Using Yamane’s technique, the blue PROLENE haptics can absorb 532 nm wavelength, resulting in the generation of heat and the creation of a flange [103]. The laser is delivered to the IOL haptic tip through the endoprobe, with the continuous delivery mode employing a power of approximately 200 mW (Fig. 4a) [103]. The haptic tip is gradually approached from a distance until flange formation is detected [103]. Laser-induced flange is smoother, more uniform and exhibits minimal charring when compared to the cautery-induced flange [103].

Then, the spirit lamps were utilized to heat the needle directly [103]. Before the invention of disposable thermal cautery, the ball cautery was heated via spirit lamp to cauterize bleeders in extracapsular cataract surgery [103]. The tip of the 26-gauge needle can also be directly heated for the same purpose (Fig. 4b) [103]. Once the tip has been heated sufficiently, the needle shaft can be employed to create a flange in a manner akin to a disposable cautery [103].

#### Four-flanged intrascleral fixation technique

To simplify and enhance the safety of scleral fixation for a single-piece nonfoldable IOL, a novel method of intrascleral sutureless fixation was created. This technique was aided by a 5–0 polypropylene suture, which was introduced using a 26-gauge needle, suitable for this purpose [93, 167]. By doing so, the issue of IOL haptic docking while using the Yamane technique was resolved, and the necessity for specific 30-gauge thin-walled needles was eliminated.

The insertion of a scleral tunnel for the intrascleral fixation of IOL (OP-72; Mediphacos, Belo Horizonte, Brazil) involved using 5–0 polypropylene sutures and cautery to form two flanges outside and two flanges in the eyelets of the IOL, without the need for flaps, knots, or glue (Fig. 3e) [93, 167]. Of note is 5–0 polypropylene, which is thicker than 10/0 and, as a result, more resistant to descent [104]. The patients were monitored for 4 months without any postoperative complications, and the IOL was centered without any tilt [93]. However, to determine its safety and efficacy, further studies with a much larger patient cohort and longer follow-ups are required [93].

## Challenges in suture and sutureless scleral fixation of IOL

### Suture fixation technique and suture selection

Improved suture fixation techniques have significantly reduced IOL instability and pupillary capture. Nevertheless, re-dislocations attributed to suture factors continue to be a challenge. The cause of these re-dislocations may be associated with suture degradation, as well as "wear and tear" of the suture by the IOL haptic and scleral tissue. The selection of suture types is of utmost importance as it determines the extent of suture degradation, fatigue resistance, and harm to the surrounding tissue.

The primary sutures used for scleral fixation are 10–0, 9–0, and 8–0 polypropylene sutures, along with Gore-Tex sutures [19]. Polypropylene sutures demonstrate physicochemical and chemical stability, high-temperature resistance, and are widely used for intraocular suturing. Clinically, 10–0 polypropylene sutures are commonly used to fix IOLs [19]. However, studies have shown that the long-term repositioning rate of IOLs fixed with 10–0 polypropylene sutures is not ideal. Vote et al. discovered that after a 60-month follow-up, 27.9% of sutures had broken, with the average sutural breakage time being approximately four years after surgery [23]. No additional studies have confirmed these findings [105]; factors such as suture degradation and surgical manipulation may contribute to suture damage [106]. Clinical observations suggest slight sutural damage at forceps-pinched locations [106]. Holland et al. proposed that the suture breakage rate may be higher than reported due to fibrosis around IOL haptics leading to stable IOLs [107].

To address issues with 10–0 polypropylene sutures, certain practitioners have utilized 9–0 polypropylene sutures for scleral fixation. Polypropylene sutures have a longer diameter (50%), greater cross-sectional area (125%), and higher tensile strength (60%) than 10–0 polypropylene sutures, suggesting superior fracture resistance [106]. The effectiveness of 9–0 polypropylene sutures for IOL fixation was demonstrated by Price et al. [106]. Another study found that the percentage of breakage was similar for 9–0 polypropylene and 10–0 sutures over an average follow-up period of 63.9 months [108]. This may be related to factors such as different stitching methods and whether the knot is wired or not.

Recently, 8–0 polypropylene sutures have increasingly been used for IOL fixation [56]. They are superior to 9–0 and 10–0 polypropylene sutures because of increased thickness and higher tensile strength [109]. In theory, 8–0 polypropylene sutures are slimmer, less bulky, and more affordable than Gore-Tex sutures [109]. John et al. have employed 8–0 polypropylene sutures for four-point scleral fixation and achieved good results [46]. Mo and Li used 8–0 polypropylene sutures in combination with the Hoffmann pocket technique to fix the IOL [110]. Postoperative complications were mostly temporary and self-limited, and no suture-related complications or IOL tilting, dislocation or subluxation were found [110]. However, the larger knots of 8–0 sutures can be difficult to bury under the scleral flap though this feature allows for intrascleral fixation. A study assessing eight different IOL fixation techniques with a minimum follow-up period of 6 months revealed that chronic glaucoma incidence was highest in the 8–0 polypropylene sutures scleral fixation group [111]. The safety and efficacy of scleral fixation with 8–0 polypropylene sutures still warrants further evaluation.

To prevent the risk of late suture breakage, Gore-Tex, a new suture commonly used in cardiothoracic surgery, was used for IOL fixation [112, 113]. Gore-Tex is a non-absorbable monofilament suture made of PTFE with several benefits, including high tensile strength, excellent visibility due to its whiteness, resistance to high temperatures, and causes minimal inflammatory response [114]. Moreover, there are no documented cases of suture degradation, rendering it one of the most suitable options for scleral fixation [114]. Postoperative visual acuity, IOL concentration, and stability demonstrated improvements in patients who received Gore-Tex sutures for fixation (Table 1) [87]. Khan et al. utilized Gore-Tex sutures with 23-gauge (mean follow-up of 325 days, 85 eyes) and 27-gauge (mean follow-up of 115 ± 70 days, 8 eyes) needles and noted no incidence of suture breakage with either gauge [114–116].

**Table 1:** Postoperative complications of scleral fixation using the Gore-Tex suture

Specific fixation technique	IOL type	No of eyes	Follow-up period	Postoperative complications rate	References
Ab externo scleral fixation	Bausch and Lomb Akreos AO60 or Alcon CZ70BD	85	Mean 325 (range 90–996) days	Hypotony 9.4% Ocular hypertension 7% VH 7% Hyphema 2% Serous choroidal detachment 2% Cystoid macular oedema 2% Corneal oedema 1%	[114]
Transscleral fixation of the capsular bag	Not given	56	Mean 35.3 (range 0.25–120) months	Posterior capsular opacification 50% IOL dislocation 5.4% Uveitis-Glaucoma-Hyphema Syndrome 1.8%	[118]
Scleral fixation	Bausch and Lomb Akreos AO60 (Rochester, NY, USA)	49	Mean 6.9 (range 0.9–29.4) months	Ocular hypertension 16.3% Hypotony 12.2% Significant persistent corneal edema (longer than 1 week) 8.2% CME 6.1% VH 4.8% IOL tilt 4.1% Hyphema 4.1%	[119]
Scleral fixation	Bausch and Lomb Akreos AO60 (Bridgewater, NJ)	30	Mean 502 ± 165 (range 365–1,095) days	*Early postoperative complicatios:* VH 10% Transient corneal edema 6.7% CME 6.7% Ocular hypertension 3.3% Hyphema 3.3% *Late postoperative complications:* CME 3.3%	[168]
Concurrent pars plana vitrectomy and scleral fixation	Bausch and Lomb Akreos AO60	27	Mean 200 ± 143 (range 33–576) days	Corneal edema 26.0% Ocular hypertension 25.9% Hypotony 7.4% CME 7.4% VH 7.4% Hyphema 3.7%	[169]
4-point sutured scleral fixation	Bausch and Lomb Akreos AO60 (Rochester, NY)	63	Mean 226.5 (range 27–883) days	CME (receiving an OCT within 3 months of surgery) 35% CME (not receiving an OCT within 3 months of surgery) 16% Transient ocular hypertension 15.9% VH 9.5% IOL tilt 4.7% Suture exposure 4.7% Persistent corneal edema 4.7% Retinal detachment 3.2%	[170]
Scleral fixation	A poly (methyl methacrylate) IOL with islets (Aurolab) of the appropriate dioptric power	100	Mean 23 months (range 12 months–5 years)	*Early postoperative complications:* VH 13% Retinal detachment 6% Transient hyphema 1% Late postoperative complications: CME 9% Epiretinal membrane 3% Bullous keratopathy 3%	[124]
4-point sutured scleral fixation	Bausch and Lomb Akreos AO60 (Bridgewater, NJ, USA)	53	Not given (the longest was ≥ 1 year)	CME 38.5% Postoperative hypotony 21.2% Corneal edema 5.8% Ocular hypertension 3.8% Macular hole 1.9% IOL dislocation 1.9% VH 1.9% Wound leak 1.9% Conjunctival cyst 1.9%	[120]
Modified small-incision technique of scleral fixation	Aspheric hydrophobic acrylic IOL (MicroPure 123, PhysIOL, Belgium)	10	Mean 396 (range 240–573) days	CME 30% Ocular hypertension 20% VH 20%	[171]
4-point sutured scleral fixation	Akreos AO60	20	The longest 6 months	Exposure of suture 40% Hypotony 15%, Ocular hypertension 10%, Transient VH 10% Retinal detachment 5% Transient lens opacification 5%	[125]
4-point transscleral fixation	Bausch and Lomb Akreos AO60	37	Mean 548.9 (range 39–1564) days	Transient ocular hypertension 27.0% Transient corneal edema 18.9% CME 18.9% Self-limited hypotension 5.4% Self-limited VH 2.7% Central retinal vein occlusion 2.7% Late retinal detachment 2.7% IOL opacification 2.7%	[172]
4-point sutured scleral fixation	Bausch and Lomb Akreos AO60 (AK; Bridgewater, New Jersey, USA)	24	The longest was 53 months	CME 4.2% Gore-Tex suture erosion 4.2%	[173]
Scleral fixation	Not given	30	The longest was 3 months	IOL tilt 16.66% Anterior uveitis 13.33% Corneal edema 13.33% Hypotony 10% IOL decentration 10% CME 10% Choroidal detachment 7% High IOP 7% VH 6.66%	[117]
Pars plana vitrectomy and 4-point scleral fixation	Akreos AO60	101	Mean 33.4 (range 12–62) months	Hypotony 12.9% Ocular hypertension 12.9% Corneal edema 8.9% CME 6.9% VH 5.9% Serous choroidal detachment 3.0% Hyphema 2.0% Retinal detachment 2.0% IOL decentralization 2.0% IOL opacification 1.0% Suture erosion 1.0% Pigment dispersion 1.0% Iris hernia 1.0%	[127]
4-point transscleral fixation	Bausch and Lomb Akreos AO60 (Bridgewater, NJ)	20	The longest was 6 months	IOL tilt 45% VH 10% Corneal edema 5% Retinal detachment 5%	[87]
Scleral fixation	Rayner 620 H (Spherical, Worthing, UK)	19	The longest was 6 months	*Short-term complications:* Corneal edema 16% IOP 11% *Long-term complications:* Increased IOP 5% Macular edema 5%	[174]

*IOL* = intraocular lens; *VH* = vitreous hemorrhage; *CME* = cystoid macular edema; *OCT* = optical coherence tomography; *IOP* = intraocular pressure

However, there are also special cases (Table 1). In the study by Rastogi et al., the IOL tilt rate is as high as 16.66% and the IOL decentration rate is as high as 10% [117]. Byrd et al. observed IOL dislocation in 2 eyes at the 8th month and 3rd year postoperatively, respectively [118]. Furthermore, Patel et al. reported an IOL tilting rate of 2/49 (4.1%) with an associated risk of scleritis [119]. It is worth noting that CME is a common postoperative complication after Gore-Tex suture fixation, with an incidence ranging from 2% to 38.5% (Table 1). In a study with a CME incidence of 38.5%, 21.2% of these eyes persisted until the last follow-up [120]. This could be attributed to the fact that optical coherence tomography macula imaging was routinely obtained during the 1-month postoperative follow-up in all patients, which was not performed in previous studies [120]. Another factor contributing to the complexity of the cases was that the study sample solely comprised patients from a tertiary care center, and approximately 40% of the cases were secondary to CME-related globe trauma [120–122]. Nonetheless, intravitreal triamcinolone acetonide prophylaxis and long-term use of nonsteroidal anti-inflammatory drugs seem to lower the likelihood of postoperative CME [123].

While Gore-Tex sutures are generally well tolerated for *ab externo* scleral fixation of IOLs, it is important to pay attention to scleral-fixation suture handling because Gore-Tex is somewhat bulky and less slippery than polypropylene [105, 114, 124]. Junqueira et al. did not rotate and bury the suture, resulting in a 40% postoperative suture exposure rate [125]. Suture exposure can lead to granuloma formation and even infection [124]. Exposure of sutures via the conjunctiva has reportedly raised the risk of contracting endophthalmitis by 24% [126].

Overall, while the Gore-Tex suture is off-label for any ophthalmic application, it shows promise as a suture fixation material with a positive short-term safety profile and clinical outcomes [112, 114]. Nevertheless, polypropylene suture fixation provided stable IOL fixation for almost 25 years, suggesting that it is still an excellent material for scleral fixation of IOLs [105]. The use of suture in patients with varying life expectancy necessitates a thorough evaluation of the potential risks and should be chosen with caution [127].

A prospective study conducted in Spain reported promising findings related to the use of nonabsorbable 10–0 polyester sutures [128]. Among 25 eyes, only one patient experienced IOL subluxation [128]. However, it is important to note that the study’s small sample size and short mean follow-up time (18.8 ± 10.9 months) limits the study’s generalizability [128].

### Sutureless fixation technique and IOL selection

Despite various improvements, intrascleral fixation presents certain issues, including postoperative complications such as pupillary capture and IOL tilt.

Pupillary capture occurs when any portion of the IOL optic is situated anterior to the iris [129]. The incidence of pupillary capture varies in clinical practice (Table 2) and can reach up to 8% following Yamane’s technique [27]. Several mechanisms have been proposed to explain pupillary capture after IOL scleral fixation, including vitreous defects, pliable iris, floppy iris, deepening of the anterior chamber, and reverse pupillary block [130]. Additionally, Choi et al. identified poor positioning of the IOL as a contributing factor [130]. Their study found that IOL decentration was significantly higher in eyes with pupillary capture compared to those without (*P* = 0.002), and recommended that symmetric fixation of the IOL can help prevent pupillary capture [130].

**Table 2:** Studies reporting IOL-related complications following intrascleral sutureless IOL fixation

Specific sutureless fixation technique	IOL type	No of eyes	Mean follow-up period	IOL-related postoperative complications rate (%) or degree (°)	Formula	References
Flanged intrascleral IOL fixation with double-needle technique	X-70 (Santen, Osaka, Japan)	50	Less than 6 months (2 eyes) More than 6 months (98 eyes)	Iris capture 2.0% IOL tilt 3.83 ± 2.69°	Not given	[27]
	ZA9003 (Abbott Medical Optics, Santa Ana, C)	32		Iris capture 15.6% IOL tilt 2.86 ± 2.18°		
	PN6A (Kowa, Tokyo, Japan)	15		Iris capture 13.3% IOL tilt 2.53 ± 1.43°		
	MA60MA (Alcon, Inc)	3		Iris capture 0% IOL tilt 5.62 ± 3.86°		
Modified Yamane technique using 27-gauge needle	IOL Master 700 apparatus (Carl Zeiss Meditec, XX, USA)	31	Minimum 6 weeks	IOL decentration 6.4%	SRK-T	[164]
Flattened flanged intrascleral IOL fixation technique	Sensar AR40 3-piece IOL (Abbott Medical Optics, Santa Ana, California, USA)	42	The longest 6 months	IOL dislocation 2.5% Iris capture 4.8%	Not given	[89]
Modified Yamane technique	Zeiss CT Lucia 602 Lens	121	Mean 237 (range 30–1079) days	Visually significant IOL tilt or decentration 5%	Holladay I, Hoffer Q, and SRK-T	[149]
Modified Yamane technique using 27-gauge needle	3-piece folded IOL (AcrySof MA60AC, Alcon or Tecnis ZA9003, AMO)	68	Mean 10 months	Iris capture 8.8% IOL tilt 2.4 ± 1.7° IOL decentration 0.35 ± 0.21 mm	Not given	[175]
Yamane flanged intrascleral haptic fixation technique	3-piece IOL	39	Minimum 3 months	IOL decentration 12.8%	SRK-T	[176]
Modified “glued” technique	MA50BM (Alcon Laboratories Inc, Fort Worth, TX) 73.7%	38	Mean 3.1 (range 1.0–5.0) years	IOL dislocation 13.2% IOL tilt 2.6%	Holladay II and Barrett Universal II	[144]
	MA60AC (Alcon Laboratories Inc, Fort Worth, TX) 18.4%					
	Unknown 7.9%					
Flanged intrascleral haptic fixation technique	CT Lucia 602 IOL (Carl Zeiss Meditec Inc, Dublin, CA) 95.5%	22	Mean 2.0 (range 1.0–3.1) years	IOL tilt 4.5%	Holladay II, Barrett Universal II and SRK-T	
	MA60AC IOL (Alcon Laboratories Inc, Fort Worth, TX) 4.5%					

*IOL* = intraocular lens

In a study by Jiang et al., no occurrences of pupillary capture were reported post-surgery due to the use of IOL haptics tilted anteriorly at a 5° angle [131]. This design moves the optic section away from the iris, significantly reducing the chance of anterior arching and subsequent pupillary capture [131]. This finding suggests that pupillary capture is also dependent on the design and type of IOL [131]. However, it has been reported that when pulling the IOL haptics, the gap between the posterior surface of the iris and the IOL could be reduced, making the 5° tilt irrelevant [132]. Therefore, achieving an accurately positioned IOL remains a significant challenge in intrascleral fixation [131].

Poor IOL positioning can result in defocus, astigmatism, and ocular coma-like aberrations, affecting the spherical equivalent in cases of severe tilt and decentration [133, 134]. An IOL tilt exceeding 5° can lead to myopic shift and oblique astigmatism [135]. The successful positioning of IOLs depends on the stability of IOLs with various haptic designs.

One-piece and three-piece IOLs are categorized based on whether the optics and haptics are integrated. The one-piece IOL, which integrates the optic and haptic, provides excellent capsular bag stability [136, 137]. The optic and haptic of three-piece IOLs are manufactured from diverse materials which, upon integration, make them susceptible to decentration and tilt following implantation [136–138]. The shape of the haptic is further divided into: open-loop and plate-haptic IOLs [75]. The width and thickness of the IOL haptic, the haptic curvature start, and the haptic–optic union are key parameters in determining the biomechanical stability of the IOLs [72]. Cabeza-Gil et al. analyzed the biomechanical stability of 144 one-piece, non-angulated, hydrophobic acrylate C-loop IOLs based on various geometrical variations [72]. The results indicate that reducing the haptic–optic union and the width of the haptics improves the biomechanical stability of the IOLs, outperforming other plate, angulated, or multipiece designs [72, 74, 139]. No incidents of pupillary capture, IOL decentration, or dislocation were noted in a trial of C-loop IOL fixation, which seems to support the findings of Cabeza-Gil et al. [75]. However, other studies have also reported similar stability between plate-haptic and C-loop IOLs [61, 140]. The conflicting findings may be related to the axial length. It appears that the C-loop IOL is less stable in patients with longer axial lengths, particularly those with myopia. This suggests a causal connection between axial length and IOL stability [141]. Hence, the type of IOLs employed are among the factors impacting the dissimilar postoperative outcomes of distinct intrascleral fixation approaches.

Kumar et al. conducted two studies on the postoperative outcome of the glued PMMA IOLs fixation technique [80, 142]. The incidence of pupillary capture was 2.63% (4/152) and 5.70% (11/191) in the respective studies [80, 142]. The incidence of IOL decentration was 1.97% (3/152) and 2.60% (5/191), respectively [80, 142]. The enhanced stability of the IOL subsequent to Yamane’s flanged technique has resulted in several advanced procedures stemming from Yamane’s technique. Unfortunately, these procedures cannot prevent IOL-associated complications, and the follow-up period is inadequate for conclusive safety assessments (Table 2). For instance, Ucar et al. utilized the Sensar AR40 three-piece IOL to establish flattened flanges, noting an observed IOL dislocation in 1 eye (2.5%) and iris capture in 2 eyes (4.8%) postoperatively [89]. The modified technique by Kelkar et al. resulted in pupil capture in 4% of patients and the need for reoperation due to IOL instability in 12% of patients [143]. Subsequently, a comparison of the two sutureless scleral fixation techniques’ long-term postoperative refractive outcomes was conducted. The incidence of IOL dislocation was higher in the ‘glued’ group (13%) compared to the ‘flanged’ group (0%) [144]. In 80% of occurrences, this was due to the breakage of the haptic–optic union [144]. A possible explanation for this is that the majority of patients in the ‘glued’ group received MA50BM or MA60AC IOLs, whereas the majority of patients in the ‘flanged’ group received CT Lucia 602 IOLs [144]. IOL haptics made from PMMA have a high incidence of intraoperative haptic kinking [145, 146]. Additionally, the haptic disinsertion power of the MA60AC model is nearly 50% lower than that of CT Lucia 602 IOLs [147]. The CT Lucia 602 is a hydrophobic acrylic three-piece lens comprising of a hydrophobic acrylic optic section and PVDF haptics [147, 148]. Although the PVDF material used in the lens is stronger and more flexible than PMMA, it is not entirely immune to deformation and kinking of the haptics during manipulation, resulting in possible tilting and/or decentration of IOLs (Table 2) [147, 148]. In addition, in a study of CT Lucia 602 lens fixation using the modified Yamane technique, postoperative decreased visual acuity due to IOL tilt or decentration occurred in only 3 eyes (2.5%) [149].

It is worth noting that another key factor for the flanged technique is the match between the size of the flanged formed by the cauterized haptics of different materials and the scleral tunnels [147]. A study was conducted to evaluate the biomechanical stability of four three-piece IOLs commonly used in the United States with the flanged technique [147]. The data strongly supports the biomechanical stability of PVDF haptics, indicating that a 1.0-mm haptic length produces the most optimal flanged result [147]. In addition, the flanged shape of PMMA haptics is contingent on the position of the forceps [150]. The results showed that the shape of flange was significantly different when the distance between the forceps and the end of haptic was 1.0 mm, 1.5 mm, and 3.0 mm, respectively [150]. It was hypothesized that the heat conducting characteristics of forceps might disrupt flange formation [150].

Recently, the FIL-SSF Carlevale lens (CIL, Soleko IOL Division, Italy), a novel IOL designed for sutureless scleral fixation, has gained attention in clinical practice [151, 152]. The CIL features several unique design elements, such as haptics angled at 5° or 10° anteriorly to reduce iris contact and lower the risk of pupillary block [153, 154]. Moreover, its opposing T-shaped harpoons enable secure self-anchoring to the sclera without the need for sutures, ensuring stable placement with minimal tilt [153, 155]. The hydrophilic material of the CIL is sufficiently flexible, allowing the anchors to be positioned beneath the conjunctiva without causing significant damage to the overlying tissue [151, 156]. Various fixation techniques have been reported, with sub-conjunctival and sutureless intrascleral fixation both yielding satisfactory results [151, 152, 157]. However, potential postoperative complications include hypotony, IOL displacement or subluxation, corneal edema, transient cystoid macular edema, scleral erosion (often associated with thinner sclera as seen in myopic eyes), and VH [158]. Some studies have identified early hypotony due to leakage from the corneal tunnel and/or scleral incision as a significant complication, with reported incidences ranging from 0% to 30% [152, 159]. Additionally, transient clouding of the CIL due to thermal shock has been documented, spontaneously resolving within a few hours, indicating the overall safety of the technique [160].

Compared to traditional methods, this approach offers the key advantage of reduced invasiveness [156]. However, further studies involving a larger patient cohort and extended follow-up periods are necessary to fully understand the unique benefits of the CIL.

In summary, intrascleral fixation is an increasingly popular procedure for IOL implantation in the absence of aphakia support. While low dislocation rates have been reported with sutureless intrascleral fixation, there is wide technical variability and limited data beyond 3 years. Due to the absence of long-term follow-up studies, it is particularly important to make an individualized clinical selection of IOL type.

## Discussions

Scleral fixation methods for PCIOL have been advanced, from conventional suture fixation to the initial sutureless fixation proposed by Gabor et al. [26]. The first flanged technique was suggested by Yamane et al. in 2017 and has since become the preferred choice for implantation of IOLs when capsular support is insufficient [27]. The optimal method for suture scleral fixation must incorporate characteristics like being simple, safe, minimally invasive, stable, and adjustable (to ensure that the optic is properly centered).

The suture fixation technique offers advantages such as conforming to ocular anatomy, broad indications, and definitive efficacy [34]. However, it is associated with potential late complications, including suture and knot erosion, degradation, and loosening, leading to IOL dislocation and tilt [41]. This is particularly concerning in children due to their longer life expectancy, continued ocular growth, and higher risk of ocular trauma [161]. Although avoiding suture knots, knotless suture fixation without a knot does not prevent suture breakage and secondary IOL dislocation. Additionally, knotless suture fixation using the transscleral Z-suture method without a scleral flap increases the risk of intraocular infection [17]. This method exposes the suture on the conjunctiva or scleral surface, thereby augmenting the danger of intraocular infection. To mitigate suture breakage, surgeons have adopted thicker sutures for IOL fixation [53, 162]. As previously stated in various studies, transscleral IOL fixation using 9–0 or 8–0 polypropylene or Gore-Tex sutures is a secure and efficient method of enhancing patient vision [87, 106, 163]. While research emphasizes the need for improving suture material and type to prevent complications, the efficacy of these approaches requires validation through long-term longitudinal studies.

Sutureless scleral fixation methods have also gained significant attention due to reduced suture-related complications, improved technical simplicity, and favorable outcomes. However, intrascleral fixation for IOLs poses challenges, including difficulty in creating a scleral tunnel and challenges in quantifying puncture points and distances, leading to unstable haptic fixation and asymmetry, resulting in late tilt and decentration of the IOL. Yamane introduced flanged intrascleral PCIOL fixation in 2017, enhancing stability with improved tunnel closure and reduced complication risks [27].

Optimizations, including the use of larger gauge needles, have shown significant visual improvements [96, 164, 165]. However, attention must be paid to fully retracting the IOL haptics into the tunnel to avoid postoperative complications such as hypotony and haptic exposure. Minor modifications to the Yamane technique aim to enhance surgeon comfort and technique, reducing tissue damage and increasing stability and safety. Improved Yamane techniques, such as those discussed by Ucar et al. [89, 90], including the facilitated trailing haptic externalization technique[91], have demonstrated benefits such as small incisions, rapid recovery, minimal complications, symmetrical haptic fixation, and accurate optic positioning, and thus is ideal for clinical use. Nevertheless, studies on the improved Yamane technique are limited, with small sample sizes and short follow-up periods hindering direct comparisons including safety profiles. Future large-scale, multi-center studies are needed to guide clinical management, particularly in IOL type selection for patients with dislocated IOLs.

## Conclusions

Looking forward, improvements in IOL fixation techniques should prioritize refining surgical techniques and materials to improve surgical efficiency, success rates, and the stability and long-term results. Innovations like robot-assisted surgery or laser technology can be introduced to improve the precision and safety. However, despite these improvements, various late complications may still arise, such as retinal detachment and proliferation of the posterior cataract capsule. Long-term outcomes of these techniques are not yet fully understood, and longer follow-up studies are necessary to assess their safety and efficacy. Furthermore, patients’ ocular anatomy and physiological characteristics differ, necessitating personalized treatment plans and customized surgical procedures. The use of advanced techniques and materials may increase the cost of surgery, limiting their widespread use.

The success of newer techniques in overcoming previous limitations has made scleral fixation more widely adopted. Therefore, this chronological evaluation underscores the need for ongoing innovation and refinement in scleral fixation techniques, moving beyond mere modifications of existing methods to embrace new approaches that enhance surgical efficacy and patient safety.

Finally, continuous technological advancements should be accompanied by an emphasis on assessing long-term outcomes and developing individualized treatment plans. The challenges must be resolved gradually through ongoing research and clinical practice.

## Data Availability

Not applicable.

## Abbreviations

IOL: Intraocular lens
PCIOL: Posterior chamber intraocular lens
CME: Cystoid macular edema
PMMA: Polymethylmethacrylate
IOP: Intraocular pressure
VH: Vitreous hemorrhage
Z-suture: Zigzag-shaped suture
logMAR: Logarithm of the minimum angle of resolution
PVDF: Polyvinylidene fluoride
PTFE: Polytetrafluoroethylene
