# Correction: Recent advances and current challenges in suture and sutureless scleral fixation techniques for intraocular lens: a comprehensive review

**DOI:** 10.1186/s40662-025-00468-8

**Published:** 2025-12-26

**Authors:** Han Sun, Caixia Wang, Hong Wu

**Affiliations:** Department of Ophthalmology, The Second Hospital of Jilin University, Changchun, China

**Correction: Eye and Vision (2024) 11:49** 10.1186/s40662-024-00414-0

Following publication of the original article [1], it was found that Fig. 2a is incorrect and Fig. 2b will serve as the revised Fig. 2, the in-text citation “(Fig. 2a)” should be deleted and all references to “Fig. 2b” should be updated to “Fig. 2.” The original paper has been updated.

The incorrect Fig. 2 is:

**Fig. 2:**
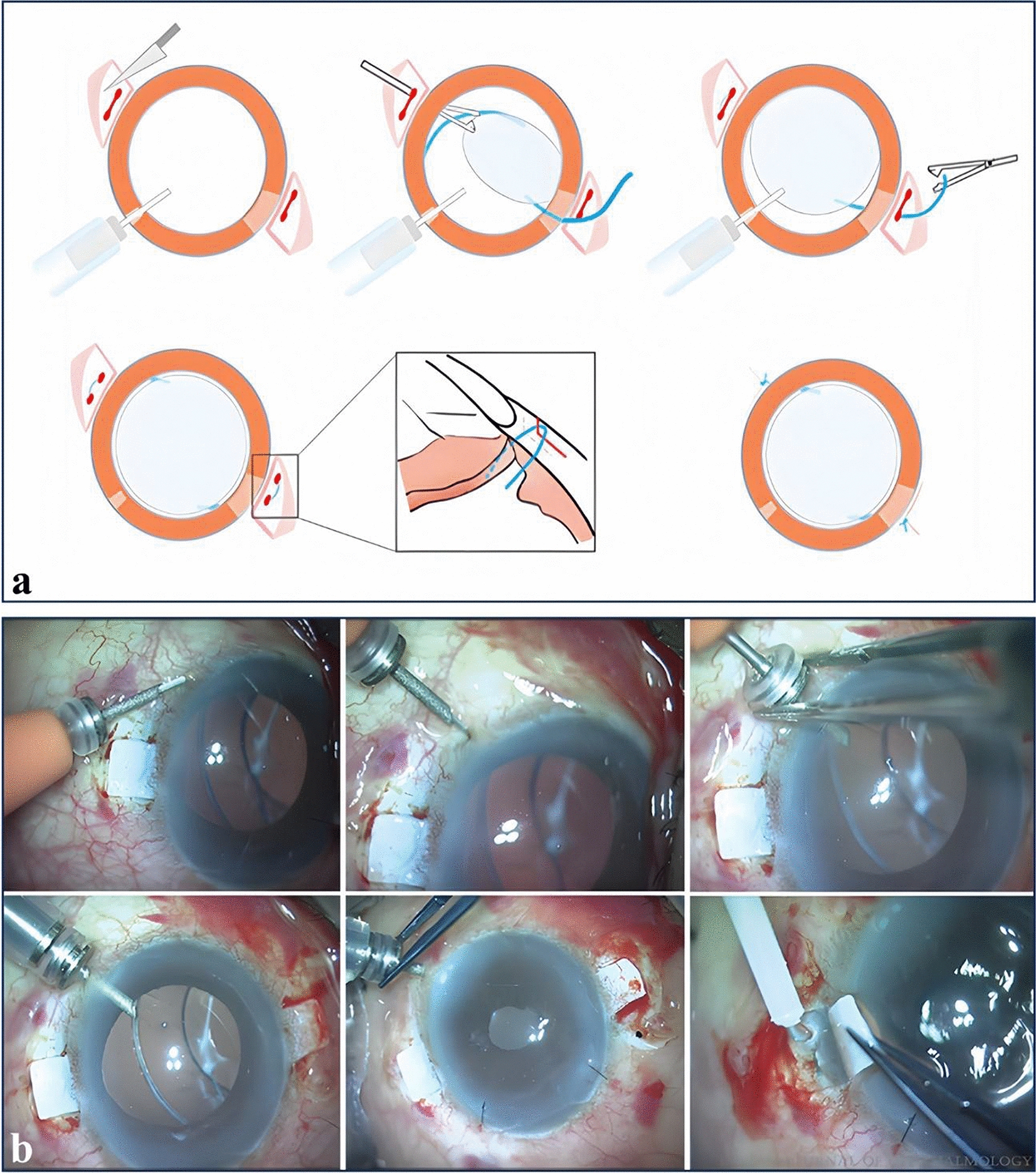
Gabor’s technique and Agarwal’s glued IOL technique. **a** Gabor’s technique. Main surgical procedures: scleral tunnel preparation, externalization, and pulling the haptic into the limbus-parallel tunnel. (Reproduced with permission from Ref. [166]). **b** Agarwal’s glued IOL technique. Fibrin glue is applied, and the flap is sealed down over the haptic. (Reproduced with permission from Ref. [98])

The correct Fig. 2 is

**Fig. 2:**
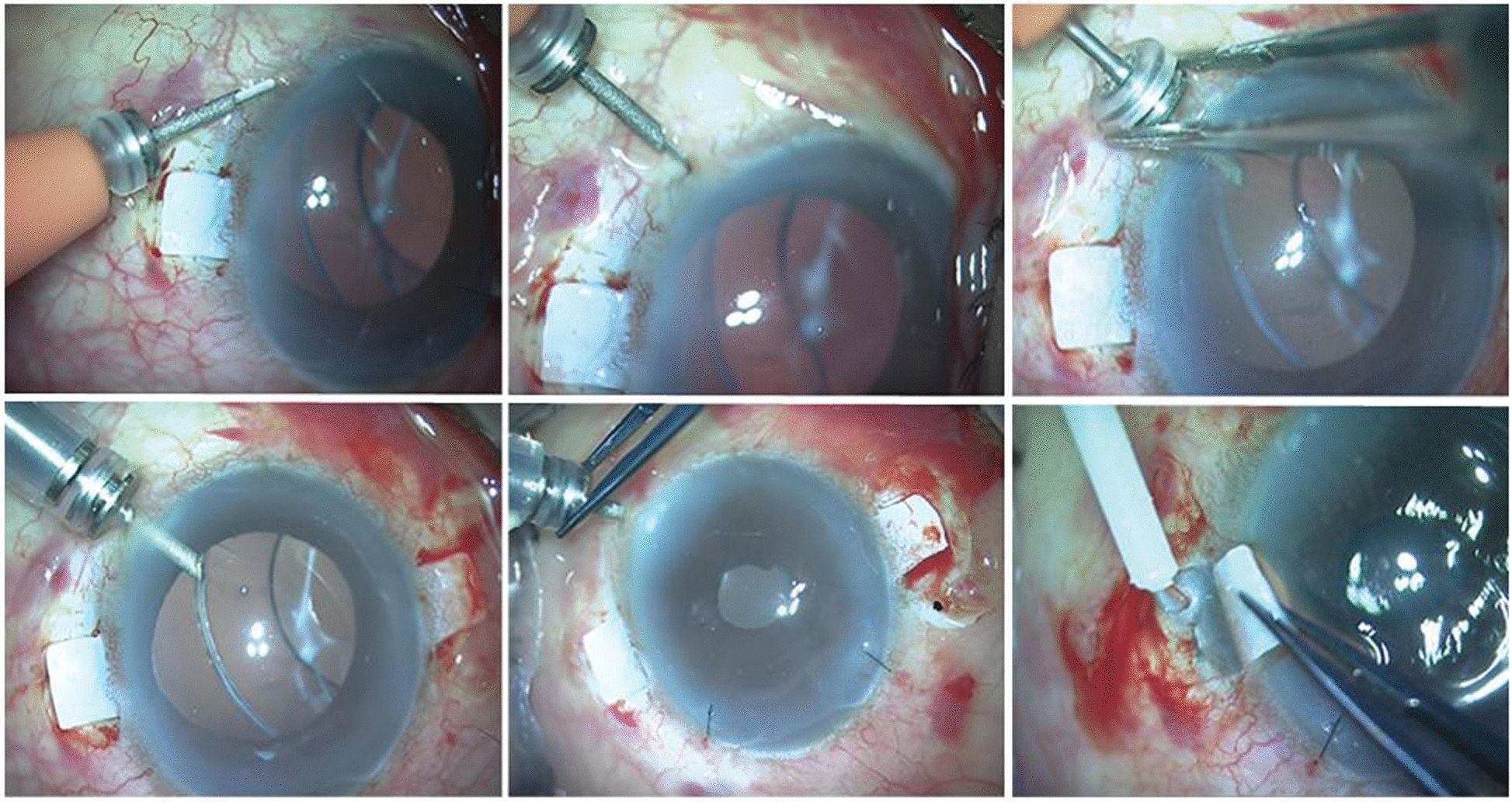
Agarwal’s glued IOL technique. Fibrin glue is applied, and the flap is sealed down over the haptic. (Reproduced with permission from Ref. [98])
